# Multiple strategies to identify HIV‐positive black men who have sex with men and transgender women in New York City: a cross‐sectional analysis of recruitment results

**DOI:** 10.1002/jia2.25091

**Published:** 2018-03-14

**Authors:** Julie Franks, Sharon B Mannheimer, Yael Hirsch‐Moverman, Eleanor Hayes‐Larson, Paul W Colson, Hugo Ortega, Wafaa M El‐Sadr

**Affiliations:** ^1^ Harlem Prevention Center ICAP at Columbia University New York NY USA; ^2^ Harlem Hospital Center New York NY USA; ^3^ Department of Epidemiology Columbia University Mailman School of Public Health New York NY USA

**Keywords:** Testing, Men who have sex with men, Transgender people, Prevention, Respondent‐driven sampling, Risk factors, Recruitment

## Abstract

**Introduction:**

Black men who have sex with men and transgender women are at high risk for HIV infection, but are more likely to be unaware of their infection or not in care for diagnosed HIV compared to other races. Respondent driven sampling has been advanced as a method to reach stigmatized and hidden populations for HIV testing. We compared strategies to recruit black, substance‐using men who have sex with men and transgender women to identify newly diagnosed HIV infection, or those previously diagnosed but not in care.

**Methods:**

The STAR (Seek, Test, and Retain) study (ClinicalTrials.gov NCT01790360) used several recruitment strategies to identify black, substance‐using men who have sex with men and transgender women with undiagnosed HIV infection or with previously diagnosed HIV infection but who were not in HIV care. Respondent‐driven sampling, community‐based recruitment and online advertising were used to recruit participants. Incentivized peer referral was integrated into all recruitment strategies. Participants completed interviewer‐administered questionnaires and HIV testing. Demographic and HIV risk‐related characteristics and recruitment strategy were summarized and stratified by HIV status. Associations were tested using Pearson's chi‐squared, Fisher's exact, and Wilcoxon rank sum tests. Factors associated with HIV‐positive diagnosis at *p* < 0.1 were included in a multivariable logistic regression model.

**Results:**

From July 2012 through October 2015, the study enrolled 1929 participants; 96.3% men who have sex with men and 3.7% transgender women. Behavioural risk factors included recent condomless anal sex (55.6%) and recent substance use during sex (73.1%). HIV prevalence was 8.7%. In multivariable analysis, significant associations with HIV infection included being transgender; non‐Hispanic black; gay/homosexual orientation; not homeless; and less likely to have insufficient income for necessities. Among recruitment strategies, respondent driven sampling was least effective in identifying HIV‐positive participants.

**Conclusions:**

Integrating multiple recruitment strategies yielded a large sample of black men who have sex with men and transgender women at substantial risk for HIV. Respondent‐driven sampling was less effective than other strategies at identifying men who have sex with men and transgender women with HIV.

## 1 Introduction

Most HIV infections in the United States (US) are reported among men who have sex with men (MSM), who represented about 2% of the population but 70% of new HIV infections in 2015 1, 2. In addition to high risk of HIV infection through anal intercourse 3, use of substances including alcohol (referred here simply as substance use) during sex, a major contributing factor to HIV risk, is prevalent in MSM across racial and ethnic groups 4, 5, 6. HIV prevalence in black MSM is markedly higher than in other racial and ethnic groups 7: recent Centers for Disease Control and Prevention (CDC) analysis estimates that one in two black MSM in the US will be diagnosed with HIV infection over the course of his life, as compared to one in four Latino and one in 11 white MSM 8. Similar to MSM, transgender women (TGW) in the US are vulnerable to HIV acquisition through anal sex 3, 9 and are severely impacted by HIV, with an estimated HIV prevalence of 28% 9, 10, 11. Available data show pronounced racial disparities among TGW populations as well 12, with an estimated 56.3% HIV prevalence in black TGW compared to 16.7% in white 13.

Differences in individual‐level risk factors do not appear to explain racial disparities in HIV prevalence among MSM 4, 5, 6, 14, 15, 16, 17, 18. Research has found that HIV‐positive black MSM are more likely to be unaware of their infection compared to white MSM 7, 19, and less likely to initiate treatment for HIV 17, 20, 21 and achieve HIV viral suppression (VS) 21, 22, which dramatically reduces HIV transmissibility 23. Black MSM are more likely than other racial and ethnic groups to report sexual partnerships with other black men; given the existing high prevalence of untreated HIV infection among black MSM, they are thus more likely than other racial and ethnic groups to be exposed to HIV through sex 24, 25, 26. Identifying black MSM and TGW with undiagnosed HIV infection and initiating treatment for them is essential to good health outcomes 27; it is also a central focus of the US strategy to control HIV and eliminate the persistent HIV disparities impacting black MSM and TGW in the US 25, 28, 29.

One of the challenges of ending the HIV epidemic in the US is reaching populations most at risk, particularly MSM and TGW. Respondent‐driven sampling (RDS), a variant of long chain referral sampling in which a cohort of purposively selected individuals (“seeds”) are enrolled and incentivized to recruit members of their social networks, was developed as a recruitment strategy that capitalizes on the power of peer influence to identify and engage members of marginalized, stigmatized, or otherwise “hidden” groups who may be less responsive to overtures from outsiders 30. The RDS approach also includes analytical methods to produce statistically valid estimations of the targeted population by accounting for non‐random influences on the likelihood of an individual being recruited, primarily the size of the peer network from which participants draw additional recruits and the prevalence within networks of given characteristics 31, 32. Studies have identified additional sources of non‐random bias that may limit the ability of RDS to produce statistically valid characterizations of the population engaged, including non‐random bias deriving from individual‐level characteristics associated with successfully recruiting members of one's social network 33; cultural factors influencing how network peers are selected for referral 34; and different ways in which recruits engage in the recruitment process 35. Several authors have concluded that RDS inference models currently available do not adequately account for these types of bias, calling into question the utility of RDS to construct valid population estimates of hard to reach subpopulations 34, 35. Nonetheless, as a recruitment method, RDS has been widely used to engage US racial and ethnic minority MSM and TGW in HIV research and testing 36, 37, 38. Some research finds RDS superior in identifying HIV‐positive 39 MSM or those at elevated risk for HIV 40 compared to the venue‐based recruitment method used in national HIV surveillance of US MSM 41, 42, in which MSM and TGW are engaged at gay‐identified commercial venues and public spaces (e.g. bars, parks). Online (OL) social and sexual networking sites offer a promising alternate venue for recruiting MSM and TGW at risk for HIV in prevention research 10, 43, 44, 45. Studies have noted differences in demographic and HIV risk characteristics among samples of black MSM identified through peer referral as compared to venue‐based methods, and conclude that combinations of recruitment methods may be more effective than a single strategy to identify HIV‐positive black MSM and TGW 40, 46, 47.

The STAR (Seek, Test, and Retain) study used several recruitment strategies to identify black, substance‐using MSM and TGW with undiagnosed HIV infection or with previously‐diagnosed HIV infection but who were not in HIV care. Here, we examine outcomes of recruitment strategies and factors associated with HIV status, including recruitment strategy.

## 2 Methods

### 2.1 Participants

MSM and TGW were eligible to participate in the study if they reported: being black; 18 years of age or older; lifetime use of illicit drugs or alcohol to intoxication; anal sex with a man in the past 12 months; and residence in New York City (NYC) with no plan to relocate during study conduct. Exclusion criteria included self‐reported prior HIV diagnosis and medical care for HIV in the past 6 months or a current prescription for antiretroviral therapy (ART), and current participation in another HIV prevention study. The study was reviewed and approved by the Columbia University Medical Center Institutional Review Board. All study participants provided written informed consent.

### 2.2 Recruitment strategies

Study recruitment was initiated using RDS procedures 48. Additional recruitment strategies were integrated approximately 12 months after initiation of RDS, to enhance enrolment and proportion of HIV‐positive participants achieved through RDS.

#### 2.2.1 RDS recruitment

Study staff were trained in RDS procedures using published training and implementation manuals 48, 49. Formative research consisting of three focus groups and eight in‐depth interviews was conducted with black MSM residing in high HIV prevalence NYC neighbourhoods to inform the demographic composition of the seed cohort, number of referral coupons (based on reported size of black MSM social networks), and level of compensation for study participation and referral of peers to the study 50. The number of study referral coupons given to each participant was set at five. During the enrolment visit, study participants were trained to use the coupons to refer peers 51. Participants were allowed one additional referral coupon to replace each of the five coupons that did not result in an enrolment, a tactic used by previous RDS studies enrolling traditionally hard‐to‐reach populations 51. Participants received $5 for every coupon that resulted in an enrolment. Two sequential cohorts of five black MSM seeds were purposively selected, based on demographic characteristics representative of black MSM at disproportionate risk for HIV infection (e.g. young 52; lower income 19) and perceived ability to refer eligible social network members (peers) into the study. Following recommendations for RDS recruitment 49, 50, enrolment was carefully monitored. Based on the enrolment of eligible participants identifying as TGW in addition to MSM, efforts were made to include a TGW seed in the planned second cohort of seeds. This seed cohort, comprising nine black MSM and one black TGW newly diagnosed with HIV infection and not in care, was subsequently enrolled in the first year of study recruitment, for a total of 20 seeds.

#### 2.2.2 Community‐based recruitment

Monitoring of enrolment during the first 12 months of RDS recruitment indicated that enrolment was both slower than expected and yielded a lower proportion of participants testing HIV positive than expected. Thus, RDS was supplemented with community‐based (CB) venue recruitment. The CB approach was informed by research showing that minority and non‐gay identifying MSM and TGW are less likely than whites and gay‐identifying individuals to be recruited at gay‐centric commercial venues such as clubs and bars 40; thus, study staff identified public spaces frequented by black MSM and TGW based on analysis of residential neighbourhoods of enrolled participants, formative research, and input from black MSM and TGW community partners. Identified venues included commercial and public transport hubs, centres of concentrated street‐based commercial sex work and areas proximate to social service providers used by black MSM and TGW. Venues were plotted on an electronic map of NYC, as were event‐specific venues such as neighbourhood gay and transgender gatherings, health fairs and other events in predominately black neighbourhoods. Outreach staff distributed recruitment material at mapped venues regularly and noted outcomes on the venues map for continual assessment of recruitment outcomes for each venue. Participants enrolled through CB recruitment were given the opportunity to refer up to five peers to the study, using the study RDS procedures. Participants subsequently enrolled with referral coupons from CB participants were identified as CB referrals.

#### 2.2.3 Online recruitment

Concurrent with the initiation of CB recruitment, the study began advertising recruitment online. Based on formative research and input from community partners, study recruitment materials were posted on one general classified advertising website, one classified advertising website oriented to MSM and TGW, and one subscription geo‐targeted social networking application used by MSM and TGW to meet male partners. In response to recruitment advertisements, prospective participants completed an online form requesting telephone eligibility screening. Staff tracked requests for screening and enrolment outcomes for online advertising. Advertising on the classified site oriented to MSM and TGW and on the subscription social networking application was halted after several months because of low response and high rates of ineligibility. Advertising on the general classified advertising site was updated weekly throughout the remaining study enrolment period. Participants enrolled through OL recruitment were given coupons to refer up to five peers, using the study RDS procedures. Participants subsequently enrolled with referral cards from OL participants were identified as OL referrals.

### 2.3 Enrolment procedures

Following informed consent, participants were given RDS recruitment cards, guidance on how to use the cards and an explanation of how incentives would be disbursed. Participants then completed interviewer‐administered questionnaires to assess demographic characteristics, substance use 53, 54, HIV risk behaviours and HIV testing history (assessed as a measure of HIV prevention service utilization). Participants were then offered rapid HIV testing via finger‐stick using OraQuick ADVANCE Rapid HIV‐1/2 Antibody Test (OraSure Technologies, Inc., Bethlehem, PA) with pre‐ and post‐test counselling. Participants received rapid test results at the enrolment visit. Those with a reactive test indicating probable HIV infection were linked to HIV care and asked to provide blood samples for confirmatory HIV testing and to measure HIV RNA (viral load). Viral load was measured using Cobas AmpliPrep/Cobas TaqMan HIV‐1 test kit version 2.0 (Roche Molecular Systems, Inc., Basel Switzerland). Participants with HIV viral load less than 200 copies/mL were considered to have VS, suggesting likely use of ART at enrolment time.

### 2.4 Data analysis

Participant characteristics and recruitment strategy were summarized and stratified by HIV status. Associations were tested using Pearson chi‐squared or Fisher's exact tests for categorical variables, and Wilcoxon rank sum tests for continuous variables. Risk factors associated with HIV status at *p* < 0.1 were included in a multivariable logistic regression model. Model fit was assessed with the Hosmer and Lemeshow Goodness of Fit test, and multicollinearity was assessed by examining variance inflation factors. All analyses were conducted, using SAS^®^ 9.4 (SAS Institute Inc., Cary, NC).

## 3 Results

**Figure 1:**
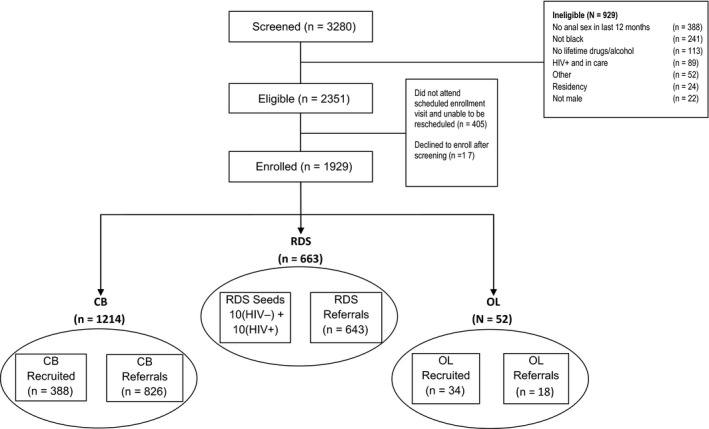
STAR recruitment flow 2012 to 2015.

A total of 3280 individuals were screened for the study (Figure 1). Of these, 929 (28.3%) did not meet study inclusion criteria. Reasons for ineligibility included: no anal sex with a man in the past 12 months (n = 388); not black (n = 241); never used drugs or alcohol to intoxication (n = 113); previously diagnosed with HIV and in care (n = 89). Of 2351 eligible individuals, 1929 (82.0%) enrolled in the study; 405 did not complete a scheduled enrolment visit and could not be contacted to reschedule. An additional 17 individuals (0.7% of those screened eligible) refused to participate in the study after screening eligible.

### 3.1 RDS seed characteristics

Participants purposively selected and enrolled as RDS seeds (N = 20) included 19 MSM and 1 TGW; all identified as black and 20% also identified as Hispanic. Median age of seeds was 27 years (IQR 23.5 to 31.5); 55% identified as gay/homosexual and 30% as bisexual. Nearly half (45%) of seeds reported frequently insufficient income for necessities in the past year and 45% described themselves as homeless at the time of enrolment. Problematic substance use in the past year was reported among 40% of seeds. Risk factors for HIV infection in the 30 days preceding enrolment among seeds included one or more casual sexual partners (70%), condomless anal sex (65%), and substance use during sex (75%). Half the seeds were purposively selected as newly diagnosed with HIV infection and not in care; HIV testing indicated the remaining 50% were HIV‐negative. Forty percent of seeds referred no peers into the study using study RDS coupons; 25% referred one and 35% referred two or more. HIV‐negative seeds referred a median of 2 (IQR 1 to 3) peers to the study; HIV‐positive seeds referred a median of 0 (IQR 0 to 1) (data not shown).

### 3.2 Enrolment by recruitment strategy

As shown in Table 1, RDS recruitment over 39 months (7/12 through 10/15) using 20 seeds yielded 643 participants, an enrolment rate of 16.5 participants per month. CB recruitment occurred over 26 months and yielded 386 CB participants and an additional 822 CB referrals (total of 1208 participants), an enrolment rate of 46.5 participants per month. OL recruitment over 26 months yielded 38 OL participants and 14 OL referrals (total of 52), an enrolment rate of 2 participants per month. A majority (62.5%) of participants referred no peers to the study using study referral coupons; 16.6% referred one and 20.9% referred two or more, as seen in Table 1. Referral of peers did not vary significantly by recruitment strategy (data not shown).

**Table 1:** Participant characteristics by recruitment strategy, excluding RDS seeds^a^

	Total^b^ (N = 1909)		RDS^b^ (N = 643)		Community‐based Recruitment (N = 388)		Community‐based Referred^c^ (N = 826)		Online (N = 34)		Online referred^d^ (N = 18)	
	n	(%)	n	(%)	n	(%)	n	(%)	n	(%)	n	(%)
HIV history												
Reactive HIV rapid test at baseline	167	(8.7%)	24	(3.7%)	47	(12.1%)	83	(10.0%)	6	(17.6%)	7	(38.9%)
No previous diagnosis	143	(85.6%)	23	(95.8%)	38	(80.9%)	70	(84.3%)	5	(83.3%)	7	(100.0%)
Virally suppressed^e^	73	(43.7%)	5	(20.8%)	17	(36.2%)	44	(53.0%)	3	(50.0%)	4	(57.1%)
Number of HIV tests in past year												
None	748	(39.3%)	240	(37.6%)	130	(33.5%)	363	(43.9%)	9	(26.5%)	6	(33.3%)
1 to 2 times	658	(34.6%)	228	(35.7%)	123	(31.7%)	288	(34.9%)	11	(32.4%)	8	(44.4%)
3 or mote times	498	(26.2%)	170	(26.6%)	135	(34.8%)	175	(21.2%)	14	(41.2%)	4	(22.2%)
Demographic characteristics												
Age, median (IQR)	42	(28 to 49)	42	(30 to 49)	34	(23 to 47)	45	(33 to 51)	30	(26 to 46)	30	(24 to 44)
Hispanic	499	(26.2%)	185	(28.8%)	103	(26.5%)	204	(24.7%)	6	(17.6%)	1	(5.6%)
Transgender	70	(3.7%)	21	(3.3%)	27	(7.0%)	21	(2.5%)	0	(0.0%)	1	(5.6%)
Sexual orientation												
Bisexual	1107	(58.1%)	415	(64.6%)	206	(53.2%)	470	(57.0%)	11	(32.4%)	5	(27.8%)
Heterosexual	239	(12.5%)	90	(14.0%)	36	(9.3%)	106	(12.9%)	5	(14.7%)	2	(11.1%)
Gay/Homosexual	487	(25.6%)	112	(17.4%)	132	(34.1%)	214	(26.0%)	18	(52.9%)	11	(61.1%)
Homeless	1034	(54.4%)	358	(55.7%)	197	(51.2%)	466	(56.7%)	5	(14.7%)	8	(44.4%)
Insufficient income for necessities												
Never	282	(14.8%)	85	(13.3%)	68	(17.6%)	109	(13.2%)	13	(38.2%)	7	(38.9%)
Occasionally	1039	(54.6%)	369	(57.6%)	193	(49.9%)	449	(54.5%)	19	(55.9%)	9	(50.0%)
Frequently	583	(30.6%)	187	(29.2%)	126	(32.6%)	266	(32.3%)	2	(5.9%)	2	(11.1%)
HIV risk factors												
Problematic substance use in the last year^g^	1346	(73.4%)	504	(78.6%)	219	(61.7%)	588	(74.9%)	23	(67.6%)	12	(66.7%)
Ever injected drugs	401	(21.0%)	156	(24.3%)	48	(12.4%)	188	(22.8%)	7	(20.6%)	2	(11.1%)
Any condomless sex in last 30 days^f^	1211	(63.5%)	393	(61.3%)	253	(65.4%)	529	(64.0%)	24	(70.6%)	12	(66.7%)
Any condomless anal sex in last 30 days^f^	1059	(55.6%)	336	(52.5%)	224	(57.9%)	467	(56.5%)	22	(64.7%)	10	(55.6%)
Any transactional sex in last 30 days^g^	1136	(59.5%)	389	(60.5%)	227	(58.5%)	485	(58.7%)	23	(67.6%)	12	(66.7%)
Substance use with sex in the last 30 days^g^	1395	(73.1%)	464	(72.2%)	295	(76.0%)	597	(72.3%)	24	(70.6%)	15	(83.3%)
Number of participants referred												
0	1193	(62.5%)	355	(55.2%)	267	(68.8%)	527	(63.8%)	31	(91.2%)	13	(72.2%)
1	317	(16.6%)	117	(18.2%)	60	(15.5%)	136	(16.5%)	2	(5.9%)	2	(11.1%)
2 or more	399	(20.9%)	171	(26.6%)	61	(15.7%)	163	(19.7%)	1	(2.9%)	3	(16.7%)

a Percentages given out of column totals excluding missing (viral load, 13; HIV testing history, 5; Hispanic, 1; transgender, 4; sexual orientation, 4; homeless, 7; education, 3; insurance, 11; insufficient income, 5; problematic drug use, 65; alcohol use, 101; alcohol or drug use, 76; social support, 287; number of partners, 5; unprotected sex, 3; unprotected anal sex, 4.

b Excludes seeds (n = 20).

c Defined as participants who had a coupon at enrolment, where the first participant in the referral chain was recruited at a community‐based venue.

d Defined as participants who had a coupon at enrolment, where the first participant in the referral chain was recruited online.

e Defined as <200 copies/mL.

f Defined as either ≥8 score on the AUDIT Test 54 or >3 on the TCU Drug Screen‐II 53.

g Participants who did not report sex in the last 30 days are coded as not having the sexual risk behaviour listed.

### 3.3 Participant characteristics

The overall study sample included 1909 participants (Table 1), excluding the purposively selected RDS seeds. Participants had a median age of 42 years; all identified as black and 26.2% identified as Hispanic ethnicity. TGW made up 3.7% of the sample. A majority (58.1%) identified as bisexual, 25.6% as gay/homosexual, 12.5% as heterosexual, and 3.8% as another sexual orientation. Current homelessness was reported by 54.5% and 30.6% reported frequently having insufficient income for necessities in the previous year. Most participants (73.4%) reported problematic substance use in the past year. Many participants reported risk factors for HIV infection within 30 days of enrolment, including condomless anal sex (55.6%), substance use during sex (73.1%), and having one or more casual sexual partners (60.6%); 27.3% of participants had a main sexual partner who was HIV‐positive or of unknown status. No HIV testing in the past year was reported by 39.3% of participants; 34.4% reported one to two tests; 26.2% reported three or more tests.

Overall, 8.7% (167 of 1909 participants) tested HIV‐positive. Among HIV‐positive participants, 14.4% reported being previously diagnosed with HIV and out of care for 6 months or longer; the remaining 85.6% reported no prior HIV diagnosis. A substantial proportion (43.7%) of HIV‐positive participants had laboratory results indicating VS at enrolment.

### 3.4 Factors associated with HIV infection

In unadjusted analysis (Table 2), characteristics associated with HIV infection included: non‐Hispanic black (*p* < 0.001), TGW (*p* < 0.001), gay/homosexual orientation (*p* < 0.001), and more than a high school education (*p* < 0.001). Participants who reported homelessness at enrolment (*p* < 0.001) and problematic substance use in the past year were less likely to be HIV‐positive (*p* < 0.001). Risk factors associated with HIV infection included condomless anal sex in the past 30 days (*p* = 0.013) and a main partner with HIV‐positive or unknown status (*p* = 0.027). The number of HIV tests in the past year reported by participants was associated with HIV status (*p* < 0.001) with proportionally more HIV‐positive participants reporting no HIV tests in the past year.

**Table 2:** Participant characteristics and unadjusted analyses of factors associated with HIV‐positive diagnosis^a^^,^ b

	Total		HIV‐positive		HIV‐negative		*p*‐value^c^
	(N = 1909)		(N = 167)		(N = 1742)		
	n	(%)	n	(%)	n	(%)	
Sociodemographic characteristics							
Age, median (IQR)	42	(28 to 49)	39	(28 to 47)	43	(28 to 50)	0.094
Black Hispanic	499	(26.2%)	21	(12.6%)	478	(27.5%)	**<0.001**
Transgender	70	(3.7%)	15	(9.0%)	55	(3.2%)	**<0.001**
Sexual orientation							**<0.001**
Bisexual	1107	(58.1%)	43	(25.7%)	1064	(61.2%)	
Gay/Homosexual	487	(25.6%)	100	(59.9%)	387	(22.3%)	
Heterosexual	239	(12.5%)	15	(9.0%)	224	(12.9%)	
Other/Don't know	72	(3.8%)	9	(5.4%)	63	(3.6%)	
Homeless	1034	(54.4%)	64	(38.8%)	970	(55.8%)	**<0.001**
Insufficient income for necessities							0.063
Never	282	(14.8%)	30	(18.1%)	252	(14.5%)	
Occasionally	1039	(54.6%)	98	(59.0%)	941	(54.1%)	
Frequently	583	(30.6%)	38	(22.9%)	545	(31.4%)	** **
Education completed							**<0.001**
Less than high school	549	(28.8%)	48	(28.7%)	501	(28.8%)	
High school	880	(46.2%)	55	(32.9%)	825	(47.4%)	
More than high school	477	(25.0%)	64	(38.3%)	413	(23.7%)	
HIV risk factors							
Problematic substance in the last year	1346	(73.4%)	95	(60.5%)	1251	(74.6%)	**<0.001**
Ever injected drugs	401	(21.0%)	31	(18.6%)	370	(21.2%)	0.417
Any condomless sex in last 30 days^d^	1211	(63.5%)	112	(67.1%)	1099	(63.2%)	0.321
Any condomless anal sex in last 30 days^d^	1059	(55.6%)	108	(64.7%)	951	(54.7%)	**0.013**
Any transactional sex in last 30 days^d^	1136	(59.5%)	102	(61.1%)	1034	(59.4%)	0.665
Alcohol or drug use with sex in the last 30 days^d^	1395	(73.1%)	127	(76.0%)	1268	(72.8%)	0.365
One or more casual partners	1156	(60.6%)	104	(62.3%)	1052	(60.4%)	0.634
Main partner(s) HIV status							**0.027**
Negative	535	(28.0%)	33	(19.8%)	502	(28.8%)	** **
Positive/Unknown	521	(27.3%)	56	(33.5%)	465	(26.7%)	** **
No main partner	853	(44.7%)	78	(46.7%)	775	(44.5%)	** **
Total number of partners, median (IQR)	2	(1 to 3)	2.0	(1.0 to 3.0)	2.0	(1.0 to 3.0)	0.076
Number of HIV tests in past year							**<0.001**
None	748	(39.3%)	103	(61.7%)	645	(37.1%)	
1 to 2 times	658	(34.4%)	48	(28.7%)	610	(35.1%)	
3 or more times	498	(26.2%)	16	(9.6%)	482	(27.7%)	
Previous HIV diagnosis, not in care	–	–	24	(14.4%)	–	–	
VS at enrolment	–	–	73	(43.7%)	–	–	
Recruitment characteristics							
Recruitment method							**<0.001**
RDS	643	(33.7%)	24	(14.4%)	619	(35.5%)	
CB	388	(20.3%)	47	(28.1%)	341	(19.6%)	
CB referred	826	(43.3%)	83	(49.7%)	743	(42.7%)	
OL	34	(1.8%)	6	(3.6%)	28	(1.6%)	
OL referred	18	(.9%)	7	(4.2%)	11	(0.6%)	
Referral of peers to study							**0.008**
None	1193	(62.5%)	123	(73.7%)	1070	(61.4%)	
1	317	(16.6%)	20	(12.0%)	297	(17.0%)	
2 or more	399	(20.9%)	24	(14.4%)	375	(21.5%)	

a Percentages given out of column totals excluding missing (HIV testing history, 5; Hispanic, 1; transgender, 4; sexual orientation, 4; homeless, 7; education, 3; insurance, 11; insufficient income, 5; problematic drug use, 65; alcohol use, 101; alcohol or drug use, 76; social support, 287; number of partners, 5; unprotected sex, 3; unprotected anal sex, 4).

b Excludes seeds (n = 20).

c Chi‐squared tests for categorical variables and Wilcoxon rank‐sum tests for continuous variables. Bold denotes *p*‐value <0.05.

d Participants who did not report sex in the last 30 days are coded as not having the sexual risk behaviour listed.

Recruitment method was associated with HIV status (*p* < 0.001) (Table 2). RDS recruitment yielded the lowest proportion of HIV‐positive participants, 3.7% (24 out of 643), and accounted for 14.4% of the HIV‐positive group. CB referral yielded 10% (83/826) HIV‐positive participants, accounting for 49.7% of all HIV‐positive participants. OL referrals yielded the smallest number, but the highest proportion, of HIV‐positive participants (7/18; 38.9%), accounting for 4.2% of all HIV‐positive participants. Across methods, HIV‐positive participants were less likely to refer any peers to the study (*p* = 0.008).

In multivariable analysis that included 1800 participants with complete data on all covariates and excluded seeds (Table 3), black non‐Hispanic ethnicity (adjusted odds ratio [aOR] 2.57; 95% CI 1.46 to 4.50); TGW (aOR 4.09; 95% CI 1.81 to 9.25); and gay/homosexual identity compared with heterosexual (aOR 3.81; 95% CI 1.96 to 7.41) were independently associated with HIV infection. HIV‐positive participants were more likely to not be homeless (aOR 1.65; 95% CI1.11 to 2.46); to report occasionally having insufficient income for necessities in the past year compared to frequently having insufficient income (aOR 1.60; 95% CI 1.01 to 2.56); and to report no problematic substance use in the past year (aOR 1.77; 95% CI 1.18 to 2.65). HIV‐positive participants were more likely to report no HIV tests in the past year (aOR = 7.56; 95% CI 4.00 to 14.29) or 1 to 2 tests in the past year (aOR = 2.86; 95% CI 1.47 to 5.53) compared with those who reported three or more HIV tests in the past year. In multivariable analysis, recruitment method was significantly associated with HIV infection: RDS was least effective in identifying HIV‐positive participants, with OL referred recruitment yielding the highest proportion of HIV‐positive participants compared with RDS (aOR 10.09; 95% CI 2.93 to 34.67).

**Table 3:** Adjusted odds ratios for factors associated with HIV‐positive diagnosis among participants in STAR^a^^,^ b

	Adjusted OR (95% CI)^c^	
	(N = 1800)	
Sociodemographic characteristics		
Age (years)	1.00	(0.98 to 1.02)
Black non‐hispanic	**2.57**	**(1.46** to **4.50)**
Transgender	**4.09**	**(1.81** to **9.25)**
Sexual orientation		
Bisexual	0.75	(0.37 to 1.48)
Gay/homosexual	**3.81**	**(1.96** to **7.41)**
Heterosexual	REF	–
Other/do not know	2.01	(0.67 to 6.05)
Not homeless	**1.65**	**(1.11** to **2.46)**
Insufficient income for necessities		
Never	1.18	(0.62 to 2.24)
Occasionally	**1.60**	**(1.01** to **2.56)**
Frequently	REF	–
Education completed		
Less than high school	1.02	(0.63 to 1.64)
High school	**0.56**	**(0.35** to **0.88)**
More than high school	REF	–
Risk factors		
No problematic substance use in the last year	**1.77**	**(1.18** to **2.65)**
Total number of partners, median (IQR)	1.01	(0.98 to 1.04)
Any condomless anal sex in last 30 days^d^	1.35	(0.90 to 2.04)
Main partner(s) HIV status		
Negative	REF	–
Positive/unknown	1.33	(0.79 to 2.25)
No main partner	1.55	(0.94 to 2.55)
Number of HIV tests in past year		
None	**7.56**	**(4.00** to **14.29)**
1 to 2 times	**2.86**	**(1.47** to **5.53)**
3 or more times	REF	–
Recruitment characteristics		
Method		
RDS	REF	–
CB	**3.03**	**(1.70** to **5.40)**
CB Referred	**2.25**	**(1.34** to **3.76)**
OL	**3.10**	**(1.05** to **9.14)**
OL referred	**10.09**	**(2.93** to **34.67)**
Referral of peers to study		
None	REF	–
1	0.60	(0.34 to 1.06)
2 or more	0.84	(0.50 to 1.42)

a Sample is 1800 participants with complete data on all covariates.

b Excludes seeds (n = 20).

c Adjusted odds ratios from multivariable logistic regression models. Bold denotes *p*‐value <0.05.

d Participants who did not report sex in the last 30 days are coded as not having the sexual risk behaviour.

## 4 Discussion

This study integrated incentivized referral of social network members, the defining feature of RDS, with CB and OL recruitment methods to enrol a large sample of black MSM and TGW with an overall HIV prevalence of nearly 9%. HIV prevalence varied by recruitment method, with substantially higher proportions of HIV‐positive participants enrolled via OL and CB recruitment and referral compared to RDS. Previous research indicated the potential of OL recruitment to reach MSM at high risk of HIV infection 55. However, MSM of color have been under‐represented in research samples recruited online 56, 57, and while the yield for HIV‐positive individuals via OL recruitment and referral was high in our study, the number of participants engaged was very small. Similar to studies finding high prevalence of HIV among MSM recruited through venue‐based recruitment 43, 58, 59, 60, our CB recruitment and referrals yielded the majority of study participants and a higher HIV prevalence than RDS. However, CB recruitment required a substantial, on‐going investment in materials and staff time to conduct regular outreach throughout the study catchment area as compared to RDS, and its success was dependent on nuanced understanding of patterns of socialization of black MSM and TGW cultivated in the formative research phase 50.

Previous studies have used RDS to enrol urban black MSM at rates of 42.7 per month in San Francisco 40, 70 enrolments per month in New York City 61, and 83 per month in Philadelphia 61. In contrast, our study achieved 16.5 enrolments per month through RDS (i.e. incentivized referrals from purposively selected seeds), a rate similar to a Chicago based study of young MSM aged 16 to 20, which achieved a rate of 11.5 enrolments per month only by adding several cohorts of additional seeds 62. Researchers have pointed to multiple factors not measured in established RDS analytic approaches that can impact on RDS recruitment, including how participants interpret eligibility criteria and apply them to peer groups 34 and the frequency with which peers receiving referral coupons redistribute them within their own networks rather than enrolling in the research 35. Detailed investigation of how RDS participants understand and operationalize incentivized peer referrals is needed to optimize RDS and will also contribute to our understanding of dynamics that shape network‐driven participation in HIV prevention research 35, 63. In addition, how seed cohorts are formed may influence the composition of resulting RDS samples. Studies have found differing HIV risk characteristics in study populations originating from seed cohorts purposively selected in community‐based settings versus seeds recruited online 64 and versus seeds who volunteer to participate in research and recruit their peers using RDS procedures 65. In our study, people who responded to CB and OL recruitment were not assessed for their potential to successfully refer their peers to the research as our purposively‐selected seeds were, yet the yield of referrals among the three groups was similar and RDS referral was less effective in engaging participants with HIV infection. A better understanding of what contributes to seeds’ successful referral of peers, and specifically of peers who do not otherwise engage in HIV research and services, would strengthen RDS and other social network‐based recruitment strategies.

Characteristics associated with HIV infection in this study included being non‐Hispanic black, TGW, and having gay/homosexual identity, reflecting the HIV epidemic in NYC 19 and the US 26. The proportion of TGW in our study (3.7%) is substantial, given that transgender individuals make up an estimated 0.5% of the New York State population 66. The recruitment of a substantial minority of TGW in this study, which relied in large part on referral through social networks suggests overlapping of MSM and TGW networks, a factor to be considered in the development of strategies to engage transgender individuals in health services and research 67, 68. Substance use during sex has been associated with sexual risk behaviours such as condomless sex and exchanging sex for money, drugs, or other goods 5, 6, 69, 70, and with increased risk of infection in MSM and TGW 71, 72. However, in this study, HIV sexual risk behaviours (condomless anal sex, substance use during sex) were not associated with HIV infection, consistent with literature finding that racial differences in HIV prevalence are not explained by commonly measured individual behavioural risk factors 14, 15, 25, 73. In contrast to studies finding that economic insecurity (e.g. living below the poverty line, insufficient income for daily meals) was associated with higher risk for HIV 74, 75, 76, this study found that HIV infection was inversely related to self‐reported homelessness and frequently insufficient income. This discrepancy may be due to differences in how economic status is measured. It may also reflect unmeasured heterogeneity within the category of self‐reported homelessness, which includes participants living in the NYC‐run shelter system where access to HIV testing and prevention services is widely available 77.

Nearly half of HIV‐positive participants in this study had VS at enrolment, suggesting that they were already on ART. No single recruitment strategy was significantly associated with VS at enrolment (data not shown). Non‐disclosure of previous HIV diagnosis among individuals presenting for testing has been observed previously 78, 79, 80, including in studies evaluating RDS methods to identify people with undiagnosed HIV 81. Non‐disclosure of previous diagnosis in the context of HIV testing and research may be motivated by financial incentives for study participation 81 and may be more prevalent in studies using RDS 63, where eligibility criteria are known and discussed among participants in the course of referral. Non‐disclosure of previous diagnosis contributes to overestimation of those unaware of their HIV infection 82 and of the effectiveness of strategies to identify new HIV cases 78. Thus it may be considered a limitation of studies that rely on self‐reported HIV status at enrolment, particularly those studies in which participants act as recruiters and communicate study eligibility criteria to potential participants**.**

Excluding those with VS, 5% of participants (56.3% of those with HIV infection) were either unaware of HIV infection or aware but out of care, highlighting the need to expand HIV testing efforts to identify black HIV‐positive MSM and TGW and engage them in HIV care and treatment 28. In the US, where a majority of new HIV infections occur in MSM 83 and a majority of MSM diagnosed with HIV are black or Hispanic 83, improving testing and linkage to services for black MSM and TGW is essential to controlling the HIV epidemic.

The study has some limitations. The amount of the incentive offered for successful peer recruitment ($5) may have been too small to adequately motivate many participants to refer potential participants. The study did not include differential incentive or “steering” schemes to encourage participants to refer peers that may be at elevated risk for HIV to the study or were under‐represented in the study (e.g. TGW, younger men) 84, and this may be considered another limitation. Previous research found no difference in uniform and conditional incentive schemes to encourage referral of peers at high risk for HIV to 81, and conditional incentives based on risk criteria that are known to participants may be perceived as either stigmatizing to those who fulfil the criteria or as discriminating against those who do not by offering them a lesser incentive. CB and OL recruitment strategies were initiated after RDS‐based recruitment was underway and formative research to develop them was not as extensive as that informing RDS, nor was the allocation of staff time and other resources to the implementation of CB and OL recruitment equivalent to support for RDS implementation. OL recruitment was potentially limited by reliance on classified advertising and paid social networking sites. Recent research from NYC shows that young black and Hispanic MSM and TGW used no‐cost social networking sites with much greater frequency than classified and paid subscription sites 85. Finally, while our participants comprised a large group reporting diverse risk factors for HIV, they may not be representative of black MSM and TGW populations overall, and the findings presented here are not generalizable.

The study has several strengths. Its adaptive, dynamic approach to recruitment utilized insights derived from RDS recruitment about physical and virtual locations of networks of black MSM and TGW in NYC to inform its CB and OL recruiting strategies. The RDS incentivized referral mechanism was extended to CB and OL strategies, retaining RDS’ ability to access peer networks while broadening the scope of recruitment to yield high enrolment. In addition to identifying individuals unaware of having HIV or aware but not in care, this combination approach also engaged a large number of black MSM and TGW at substantial risk for HIV who are appropriate for intensive prevention efforts. Multiple recruitment strategies were implemented in the same population over a single period of time, allowing for close comparisons among the resulting enrolled groups. Finally, while the study did not perform testing for the presence of antiretroviral drugs in the blood among participants, the study included viral load measurement among HIV‐positive participants, with use of VS as a proxy measure for engagement in HIV care and treatment to address the recognized weakness in previous research that did not differentiate between previously and newly diagnosed cases of HIV infection 78, 82.

In conclusion, this study examined factors associated with HIV infection among black, substance‐using MSM and TGW and highlights some of the challenges of engaging this important population in HIV services. Distinct recruitment strategies yielded different proportions of HIV‐positive participants, and combining RDS’ peer referral mechanism with other recruitment strategies was more successful in identifying individuals with HIV than RDS alone.

## Competing interests

The authors declare they have no competing interests.

## Authors’ contributions

JF, SBM, YHM, PWC and WME: study design, data interpretation, writing; EHL: data analysis, data interpretation, writing; HO: data analysis. All authors have read and approved the final version.

## Funding

The research reported here was supported by the National Institute on Drug Abuse of the National Institutes of Health (R01DA032100‐04). EHL was supported by the National Institute of Allergy & Infectious Diseases of the National Institutes of Health (T32AI114398) and the National Institute of Mental Health of the National Institutes of Health (T32 MH013043‐44), and YHM was supported by the National Institute of Allergy & Infectious Diseases of the National Institutes of Health (1K01A104351).
